# Removal of crystal violet from water by magnetically modified activated carbon and nanomagnetic iron oxide

**DOI:** 10.1186/s40201-015-0156-4

**Published:** 2015-01-31

**Authors:** Soheila Hamidzadeh, Marzieh Torabbeigi, Seyed Jamaleddin Shahtaheri

**Affiliations:** Payam Noor University of Varamin, Qarchak, Varamin, Tehran Iran; Faculty of Health, Safety and Environment, Shahid Beheshti University of Medical sciences, P. O. Box 16858–116, Tehran, Iran; Department of Occupational Health Engineering, School of Public Health, Institute for Environmental Research, Tehran University of Medical Sciences, Tehran, 14155-6446 Iran

**Keywords:** Magnetically modified activated carbon, Crystal Violet, Langmuir isotherm, Freundlich isotherm, Nano magnetic iron oxide

## Abstract

Magnetically modified activated carbon, which synthesized by nanomagnetic iron oxide, was used for fast and effective removal of Crystal Violet from aqueous solutions. The scanning electron microscopy (SEM) images of nano-adsorbent showed that the average sizes of adsorbent are less than 100 nm. The various parameters, affecting on adsorption process, were examined including pH and temperature of dye solution, dose of adsorbent, and contact time. Then, thermodynamic parameters of sorption were calculated. Langmuir and Freundlich isotherms were used to fit the resulting data. Adsorption kinetics was consistent with a pseudo second order equation. Thermodynamic parameters of adsorption, ∆H^0^, and ∆S^0^ were calculated. Also, for further investigations, nano magnetic iron oxides was synthesized and used as adsorbent. Sorption capacities were depending on the temperature varied from 44.7 to 67.1 mg/g and from 12.7 to 16.5 mg/g for magnetically modified activated carbon and nanomagnetic iron oxide, respectively.

## Introduction

Large amounts of dyes are produced and applied in various industries. Small amounts of dyes (less than 1 ppm for some dyes) are visible in water [1,2]. As the most of the dyes in wastewater are stable to light and oxidation and also resistant to aerobic digestion, they damage to the aquatic life [3].

Crystal Violet (CV) is a synthetic basic cationic dye used for various purposes including biological stain, dermatological agent, veterinary medicine, additive to poultry feed to inhibit propagation of mold, intestinal parasites, and textile dyeing industries etc. [4,5]. It is a mutagen, mitotic poison, and also proven potent carcinogen [6,7].

Various processes were developed for the dye removal from the wastewater including adsorption and biosorption [8-10], chemical and electrochemical oxidation [11-13], membrane separation process [14], photodegradation [15], etc.

Magnetic separation techniques have found important applications in environmental technology. In adsorption processes, the magnetic adsorbent can be easily separated from solution after adsorption process [16]. The magnetizations of adsorbents such as peanut husks [17], sawdust [18], baker's yeast cells [19], activated coconut shell carbon [20] etc. were investigated for removal dyes and other concomitances. Since Activated carbon is one of most useful adsorbent for removal of dye, in this study, it was modified by nanomagnetic iron oxide for fast and effective removal of Crystal Violet. The SEM images indicated the sizes of adsorbent particles are in nano scales. Adsorbent efficiency in removal Crystal Violet was studied. The affecting parameters on adsorption process were examined. The thermodynamic and kinetic adsorption parameters of Crystal violet onto magnetically modified activated carbon were obtained while; they have not been reported in previous studies. In order to comparative studies, nanomagnetic iron oxide (that used for magnetization of activated carbon) were synthesized and used as adsorbent.

## Materials and methods

### Reagents

Activated charcoal was purchased from BDH Ltd Poole England. Crystal Violet dye was from Merck Darmstadt Germany. All other chemicals used in this study were of high purity and used without further purification. Double distilled water was used for all experiments.

### Adsorbent

**Figure 1:**
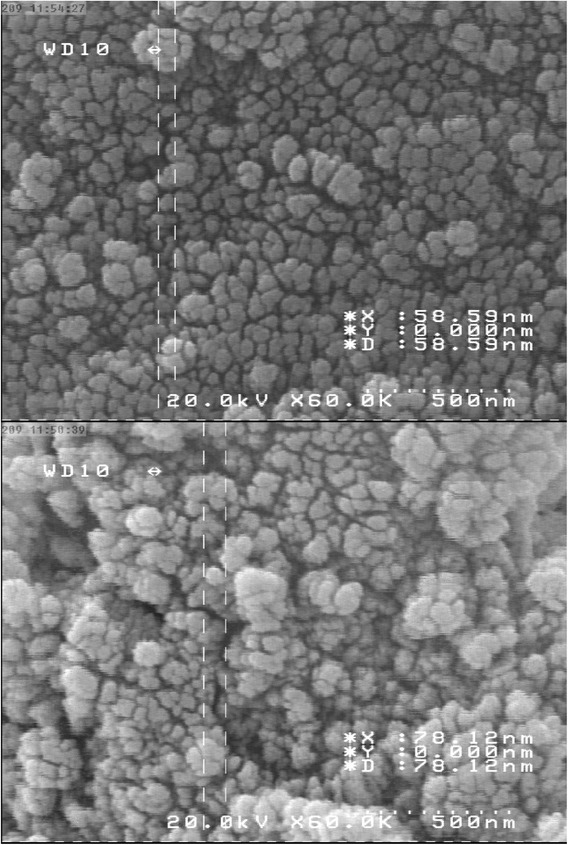
**The SEM images of synthesized magnetically modified activated carbon.**

Magnetically modified activated carbon was synthesized with slightly modified procedure described in reference [21]. 1 g of activated charcoal was placed in contact with 150 ml of sodium hydroxide 0.5 mol/l for 30 min and mixed up by stirrer thoroughly. Then, it was heated to boiling. 50 ml of solution containing Fe(NO_3_)_3_ 0.2 M and FeSO_4_ 0.1 M was added quickly. The mixture was transferred into a distillation flask to reflux for two hours. Magnetically modified activated carbon was collected by magnet and then washed by water, methanol, and acetone several times to remove excess sodium hydroxide. It was then placed in oven at 80°C to drying for 12 h. The scanning electron microscopy (SEM) images of magnetically modified activated carbon have been shown in Figure 1 indicated that the sizes of synthesized magnetic particles are less than 100 nm.

### Adsorption procedure

Batch adsorption experiments with 10 ml of Crystal Violet solution (5 mg/l) were done for 1–10 mg of adsorbent, 1 to 10 min contact times, 3–9 pHs, and at 20, 30 and 40°C.

pHs of solutions were adjusted by expected values of nitric acid and sodium hydroxide solutions. Analysis of dye concentration was carried out by UV–vis spectrophotometer in 593 nm wavelength.

Percentage of dye removal from solution was calculated by the following equation:

1 $$ Removal\left(\%\right)=\frac{C_0-{C}_i}{C_0}\times 100 $$

C_0_ and C_i_ are initial and final concentration of Crystal Violet solutions, respectively. The following formula is used to calculate the amount of dye adsorbed by the adsorbent:

2 $$ {q}_e=\left({C}_0-{C}_e\right)\times \frac{v}{w} $$

Where q_e_ is adsorption capacity (mg of adsorbed dye per g of adsorbent), C_e_ is equilibrium concentration of dye (mg/l), v is the volume of the solution (l) and w is the mass of adsorbent (g).

### Kinetic study

Pseudo-first and pseudo-second order models were applied for the adsorption kinetic studies. The first order rate equation of Lagergren was used for the adsorption:

3 $$ \log \left({q}_e-{q}_t\right)= \log \left({q}_e\right)-\frac{k_1}{2.303}t $$

Where q_e_ and q_t_ are masses of dye adsorbed at equilibrium and at time t (mg g^−1^), respectively, and k_1_ is the first-order reaction rate constant (min^−1^). Pseudo-second order equation was used based on Ho-Mckay equation:

4 $$ \frac{t}{q_t}=\frac{1}{k_2{q}_e^2}+\frac{1}{q_e}t $$

Where k_2_ is the second order reaction rate equilibrium constant (g mg^−1^ min^−1^).

## Result and discussion

### Optimization

#### The adsorbent dosage

**Figure 2:**
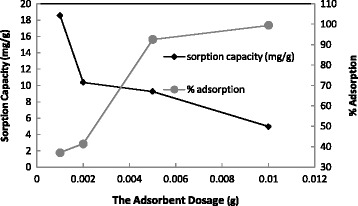
**Effect of adsorbent dosage on adsorption percentage.**

As the dose of adsorbent can strongly affect the sorption capacity, the adsorption procedure was done with varied dose of adsorbent (1–10 mg). Obtained results shown in Figure 2 indicated that, increasing amount of the adsorbents increases the contact surface area and exchangeable sites, and then increases the percent removal of dye.

#### The contact time

**Figure 3:**
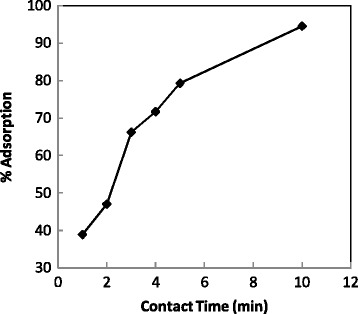
**Effect of contact time.**

The contact time is one of the important factors affecting on batch adsorption process, therefore, contact time from 1 to 10 min was studied for removal of dye. Resulting data were shown in Figure 3. This synthesized nano magnetic adsorbent removed Crystal Violet very fast, so that, more than 90 % of dye was removed in 10 min and adsorption process attains saturation at this time.

#### pH of Crystal Violet solution

**Figure 4:**
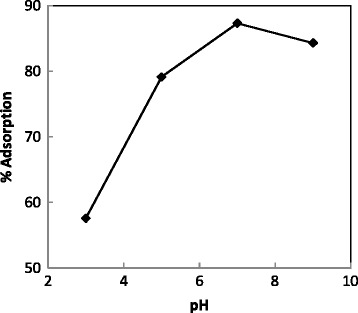
**Effect of pH.**

To study the effect of pH on the adsorbent, the range of pH adjusted between 3 and 9. The experimental date indicated that, the removal of dye was so effective at pH 9. Figure 4 shows the effect of pH on removal of Crystal Violet. Cationic dyes like Crystal Violet were adsorbed on activated carbon surface at alkaline pHs [22,23].

#### Temperature of dye solution

Dye removal was examined at different temperatures range started from 27°C (as ambient temperature) to 70°C. 10 ml of dye solution 5 mg/l was contacted to 0.01 g of magnetically modified activated carbon for 5 min at pH 5 at 27, 40, 50, 60, and 70°C.

**Figure 5:**
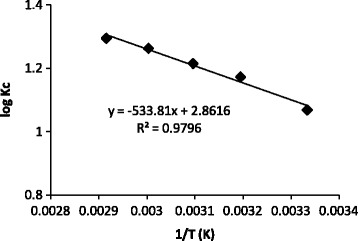
**Eyring’s plot of adsorption process.**

Thermodynamic parameters, ∆S^0^ and ∆H^0^ were calculated from Eyring’s plot; log Kc versus 1/T (Figure 5).

5 $$ \log {K}_C=\frac{\Delta {S}^0}{2.303R}-\frac{\Delta {H}^0}{2.303R}\frac{1}{T} $$

6 $$ {K}_C=\frac{C_{ad}}{C_e} $$

Where K_C_ is the equilibrium constant, C_ad_ and C_e_ are the equilibrium concentrations of the dyes on the adsorbent and in solution, respectively, T is the temperature in Kelvin and R is gas constant. Then, ∆G^0^s in different temperature were determined by:

7 $$ \Delta {G}^0=-2.303\ R\ T\; \log {K}_C $$

Obtained thermodynamic parameters are presented in Table 1. The negative values of free energy (∆G^0^) indicate the feasibility of dye removal and spontaneous nature of adsorption process. Value of ∆H^0^ confirms that the adsorption of Crystal Violet on magnetically modified activated carbon is endothermic process. The positive ∆S^0^ demonstrate the affinity of Crystal Violet on to nano adsorbent.

**Table 1:** **The thermodynamic parameters of adsorption of Crystal Violet on magnetically modified activated carbon**

**Temp. (K)**	**∆G** ^**0**^ **(kJ/mol)**	**∆S** ^**0**^ **(kJ/mol.K)**	**∆H** ^**0**^ **(kJ/mol)**
300	−6.14	0.055	10.22
313	−7.03		
323	−7.51		
333	−8.05		
343	−8.50		

### Kinetic study

**Figure 6:**
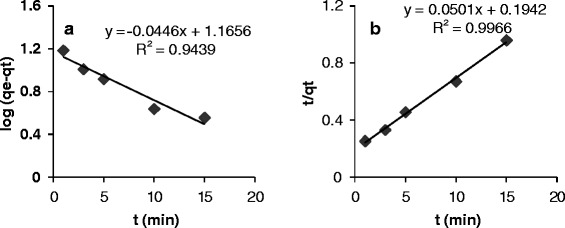
**The fitting data of adsorption procedure by (a) pseudo-first order and (b) second order equations.**

In order to determine the kinetic of adsorption, the results were evaluated by equations (3) and (4) that related to the pseudo-first order and second order kinetics, respectively. Figure 6a and b show the fitting data by pseudo-first order and second order equations. The rate constants and linear regressions (R^2^) were reported in Table 2. The results indicated that, the adsorption of Crystal Violet on magnetically modified activated carbon has been described by pseudo-second order equation.

**Table 2:** **The rate constants and linear regressions of First order and Second order for adsorption of Crystal Violet on magnetically modified activated carbon and nanomagnetic iron oxide**

***Adsorbent***	**First order**		**Second order**	
	**k**_**1**_ **(min** ^**−1**^ **)**	**R** ^**2**^	**k**_**2**_ **(g mg** ^**−1**^ **min** ^**−1**^ **)**	**R** ^**2**^
**Magnetically modified activated carbon**	0.103	0.944	0.752	0.997
**Nanomagnetic iron oxide**	0.230	0.901	1.38 × 10^−2^	0.962

In order to perform further investigation, nanomagnetic iron oxide was synthesized as similar as described in experimental section, without any addition of activated carbon. In the same conditions, nanomagnetic iron oxide was used as adsorbent. The kinetic results shown in Table 2 indicated that, the kinetic of adsorption was the same as magnetically modified activated carbon with lower rate constant (k_2_).

### Adsorption isotherms

Adsorption isotherms are important to describe the adsorption mechanism and to determine the maximum adsorption capacity and also to consider the feasibility of the application process. Langmuir and Freundlich equations were used to study the adsorption isotherms. The Langmuir model is describing the monolayer adsorption onto a surface with a finite number of identical sites. Linear form of Langmuir isotherm is given by:

8 $$ \frac{1}{q_e}=\frac{1}{q_{max}\ {K}_L\ {C}_e}+\frac{1}{q_{max}} $$

**Figure 7:**
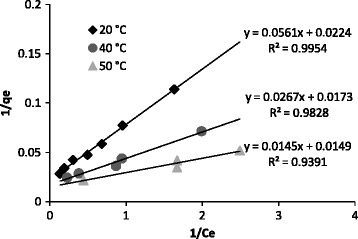
**Langmuir isotherms in various temperatures.**

Where q_max_ (mg/g) is the maximum adsorption capacity and K_L_ is a constant factor related to the energy. The variations of 1/q_e_ at different temperatures are plotted versus 1/C_e_ in Figure 7.

The Freundlich model is based on the sorption onto a heterogeneous surface. The linear expression of Freundlich equation is as follow:

9 $$ \log \left({q}_e\right)= \log \left({K}_F\right)+\frac{1}{n} \log \left({C}_e\right) $$

**Figure 8:**
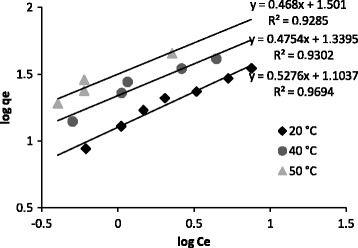
**Freundlich isotherms in various temperatures.**

Where K_F_ and n are the Freundlich constants, being indicators of adsorption capacity and adsorption intensity, respectively. Log q_e_ at different temperatures versus log C_e_ is plotted in Figure 8.

The regression coefficients and model parameters for Langmuir and Freundlich isotherms are tabulated in Table 3. The regression coefficients indicated that the data are better fitted by the Langmuir model. The maximum sorption capacity (q_max_) and intensity of sorption (K_L_) increased by arising the temperature.

**Table 3:** **The parameters of Langmuir and Freundlich isotherms at different temperatures for adsorption of Crystal Violet on magnetically modified activated carbon and nanomagnetic iron oxide**

***Adsorbent***	**Temp. (°C)**	**Langmuir**			**Freundlich**		
		**q**_**max**_	**K**_**L**_	**R** ^**2**^	**K**_**F**_	**n**	**R** ^**2**^
**Magnetically modified activated carbon**	20	44.7	0.40	0.995	12.7	1.90	0.969
	40	57.8	0.65	0.983	21.9	2.10	0.930
	50	67.1	1.03	0.939	31.7	2.14	0.929
**Nanomagnetic iron oxide**	20	12.7	0.045	0.956	8.2	1.02	0.918
	40	14.5	0.047	0.982	10.1	1.03	0.935
	50	16.5	0.051	0.970	15.5	1.19	0.952

In the same conditions, the experiments were performed by nanomagnetic iron oxide as the adsorbent. The obtained results listed in Table 3 show that, Langmuir and Freundlich did not well describe the adsorption isotherm model. The values of q_max_ increased by increasing the temperature and also were less than those for magnetically modified activated carbon.

Magnetically modified activated carbon is a perfect and effective adsorbent that offers a fast removal of Crystal Violet from water samples. The kinetic of the adsorption is pseudo second order. Langmuir isotherm can describe well the adsorption model. The sorption capacity and intensity of sorption are enhanced by increasing the temperature. Using nanomagnetic iron oxide as adsorbent compared with magnetically modified activated carbon showed that, it is able to sorption of Crystal Violet with less effectiveness. The previous studies on magnetic adsorbents for removal of Crystal Violet have been presented in Table 4. The time of removal in this study (10 min) was very short than the contact time in previous studies so that the minimum time of process for these studies with high sorption capacities was 90 min. Thermodynamic and kinetic parameters were not calculated in previous studies. Nanomagnetic iron oxide was not used for removal Crystal Violet previously.

**Table 4:** **The previous studies on magnetic adsorbents to removal Crystal Violet**

**Adsorbents**	**q**_**max**_	**Ref.**
Magnetic charcoal	10 mg cm^−3^	[23]
Magnetically labeled Baker's yeast cells	85.9 mg g^−1^	[19]
magnetically modified *Saccharomyces cerevisiae* subsp. uvarum cells	41.7 mg g^−1^	[24]
Ferrofluid modified sawdust	51.16 mg g^−1^	[14]
magnetically modified *Chlorella Vulgaris* cells	42.91 mg g^−1^	[25]
Magnetic fluid modified peanut husks	80.9 mg g^−1^	[17]
Magnetically modified spent grain	40.2 mg g^−1^	[26]
Magnetic carbon-iron oxide nanocomposite	81.70 mg g^−1^	[20]
Magnetically modified spent coffee grounds	68.1 mg g^−1^	[27]
Magnetically modified activated car bon	67.1 mg g^−1^	Present study
Nanomagnetic iron oxide	16.5 mg g^−1^	Present study

## Conclusion

The results indicate that magnetically modified activated carbon have considerable potential for the removal of Crystal Violet, also the magnetic adsorbent can be simply removed from solution by using magnet or appropriate magnetic separator after adsorption process. The obtained results of this investigation implicate that this adsorbent was more able to remove dye in the less time with compared to some of studies listed in Table 4. Also we investigated Thermodynamic and kinetic studies for removal process more than other pervious works.

The resulting adsorption capacities demonstrate that, although nanomagnetic iron oxide can be as an adsorbent, but its efficiency is much lower than magnetically modified activated carbon.
